# Surgical Management of Intestinal Obstruction Secondary to Barium Impaction: Two Exemplary Cases

**DOI:** 10.7759/cureus.76301

**Published:** 2024-12-24

**Authors:** Md Sibat Noor, Helen Liu, Boris Joutovsky, Dmitriy Rybitskiy, Gerard A Baltazar

**Affiliations:** 1 General Surgery, New York University (NYU) Langone Hospital—Long Island, Mineola, USA; 2 General Surgery, New York University (NYU) Grossman Long Island School of Medicine, Mineola, USA; 3 Surgery, New York University (NYU) Grossman Long Island School of Medicine, Mineola, USA; 4 Surgery, New York University (NYU) Langone Health/New York University (NYU) Winthrop Hospital, Mineola, USA

**Keywords:** barium obstruction, colonic bezoar, colon obstruction, ex-lap, gastric bezoar

## Abstract

A "barium chemobezoar" or "barolith" is a rare but serious cause of intestinal obstruction. We present two cases, a 70-year-old female patient and a 61-year-old male patient, both requiring urgent surgery for barolith-induced bowel obstruction. Diagnostic challenges were encountered in both cases, with imaging raising suspicion for barolith formation after prior barium use. Surgical intervention, including colotomy and enterotomy, was necessary due to the risk of perforation and ischemia. These cases highlight the variability in presentation and the lack of standardized guidelines for diagnosis and management, emphasizing the need for heightened clinical awareness and timely imaging.

## Introduction

A “barium chemobezoar” or “barolith” refers to an inspissated mixture of barium and fecal matter that forms after the oral or rectal administration of barium sulfate contrast. Although rare, barium chemobezoars can result in bowel obstruction if the volume of inspissated material occludes the bowel lumen [1]. Diagnosis is usually made using plain film or computed tomography (CT). Depending on the location, further complications reported in the literature include acute appendicitis, intussusception, ulceration, perforation, or volvulus. In cases of barolith-induced bowel obstruction, non-operative management is usually futile, and surgical intervention to evacuate the chemobezoar becomes the definitive treatment modality. We describe two cases of bowel obstruction secondary to barolith, both requiring operative intervention, and we discuss the distinctive features and opportunities to expeditiously identify and manage this disease process.

## Case presentation

Case 1

A 70-year-old woman initially presented to an outside hospital (OSH) after a mechanical fall, sustaining bilateral distal radius fractures. While at the OSH, she developed supraventricular tachyarrhythmia and was transported to our Level 1 Trauma Center for cardiac catheter ablation prior to open reduction and internal fixation of her wrists. The patient’s past medical history included hypertension, asthma, scoliosis, and laparoscopic cholecystectomy.

**Figure 1:**
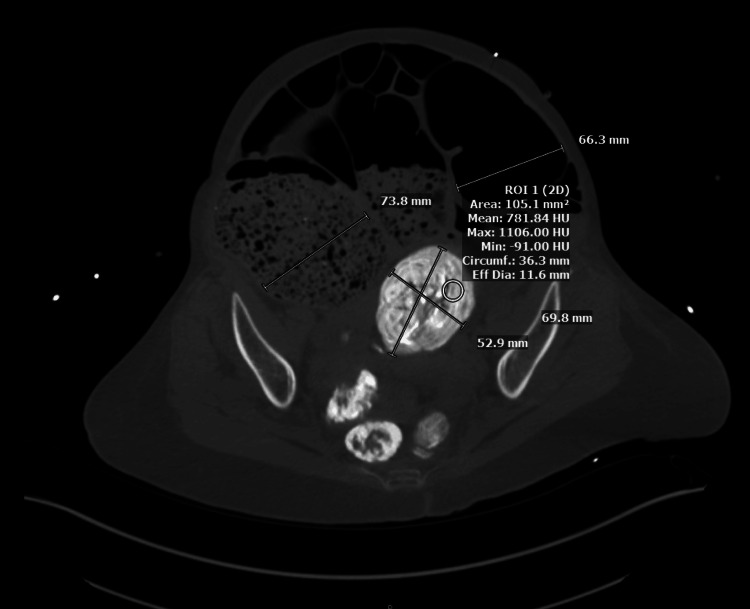
CT imaging in the axial plane revealing a large barolith lodged in the sigmoid colon with upstream dilation of the cecum and transverse colon, and the distal sigmoid is collapsed

**Figure 2:**
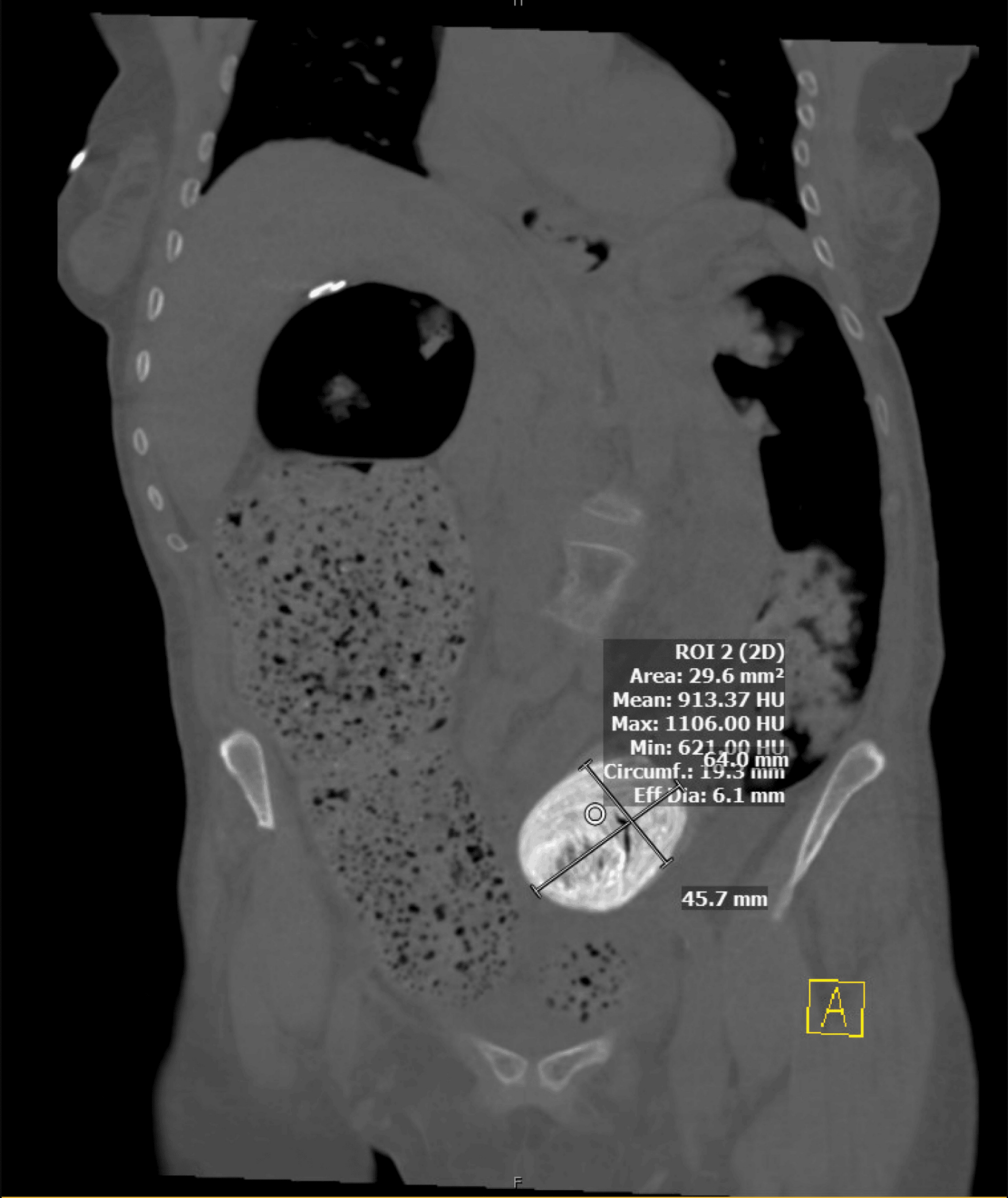
CT imaging in the coronal plane revealing a large barolith lodged in the sigmoid colon with upstream dilation of the cecum and transverse colon, and the distal sigmoid is collapsed

Upon arrival at our institution, she complained of abdominal distention and constipation. A CT scan of the abdomen and pelvis with oral and intravenous contrast revealed distention of the cecum and transverse colon, with a transition zone in the sigmoid colon from an ovoid intraluminal mass measuring 7x5 cm (Figures 1, 2). There was no radiographic evidence concerning for ischemia or perforation. Due to the cecum measuring 11 cm with a clear transition point, a decision was made to take the patient to the operating room for laparotomy. Pre-operatively, the patient underwent fluid resuscitation in the ICU, and further history revealed she had received oral barium as part of a constipation workup at the OSH.

**Figure 3:**
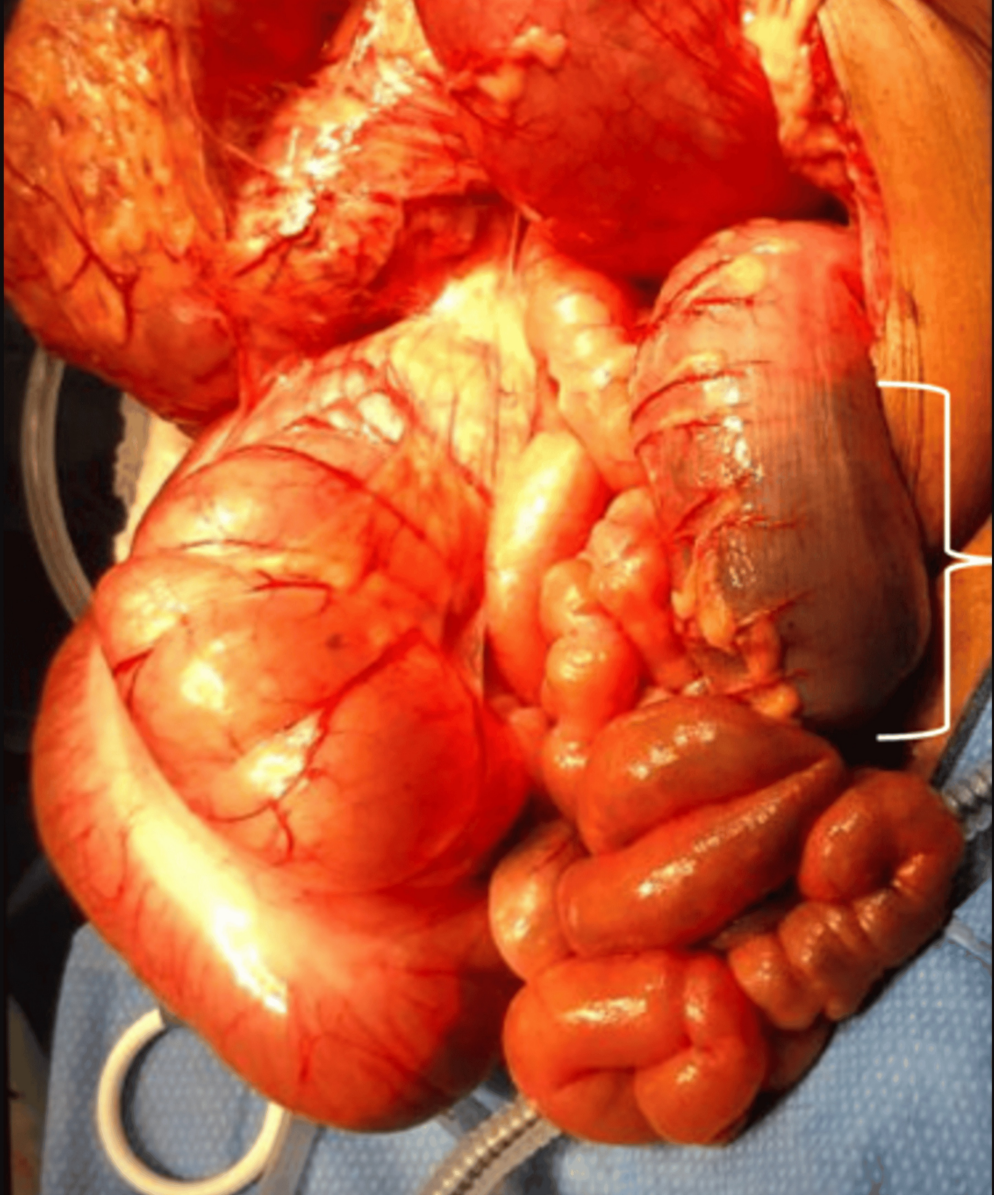
Sigmoid colon obstruction with barolith stool ball indicated by the bracket

In the operating room, the cecum was friable and significantly dilated, but there were no overt signs of bowel ischemia (Figure 3). A longitudinal colotomy, six centimeters in length, was made along the sigmoid taenia. The proximal colon was decompressed with evacuation of a large, firm barolith. The colotomy was closed transversely, hand-sewn in two layers: the first with absorbable polyglactin and the outer with silk. The patient was returned post-operatively to the ICU for continued hemodynamic monitoring. She progressed rapidly, with a return of bowel function, and was discharged on a low-residue diet on post-operative day three.

Pathology reported a 6.5 x 6.3 x 5.5 cm ball of dark brown-green solid fecal material consistent with a barium chemobezoar.

Case 2

A 61-year-old man with a history of opioid abuse-related chronic constipation, heart failure, chronic obstructive pulmonary disease, and a recent admission for intoxication and respiratory failure presented to the emergency department (ED) with 48 hours of diffuse abdominal pain and distention. The patient had seen his primary care physician (PCP) six days prior to presentation. A modified barium swallow (MBS) study to evaluate for dysphagia and aspiration risk post-intubation had been performed as part of that PCP’s workup. Notably, no increased aspiration risk was detected.

**Figure 4:**
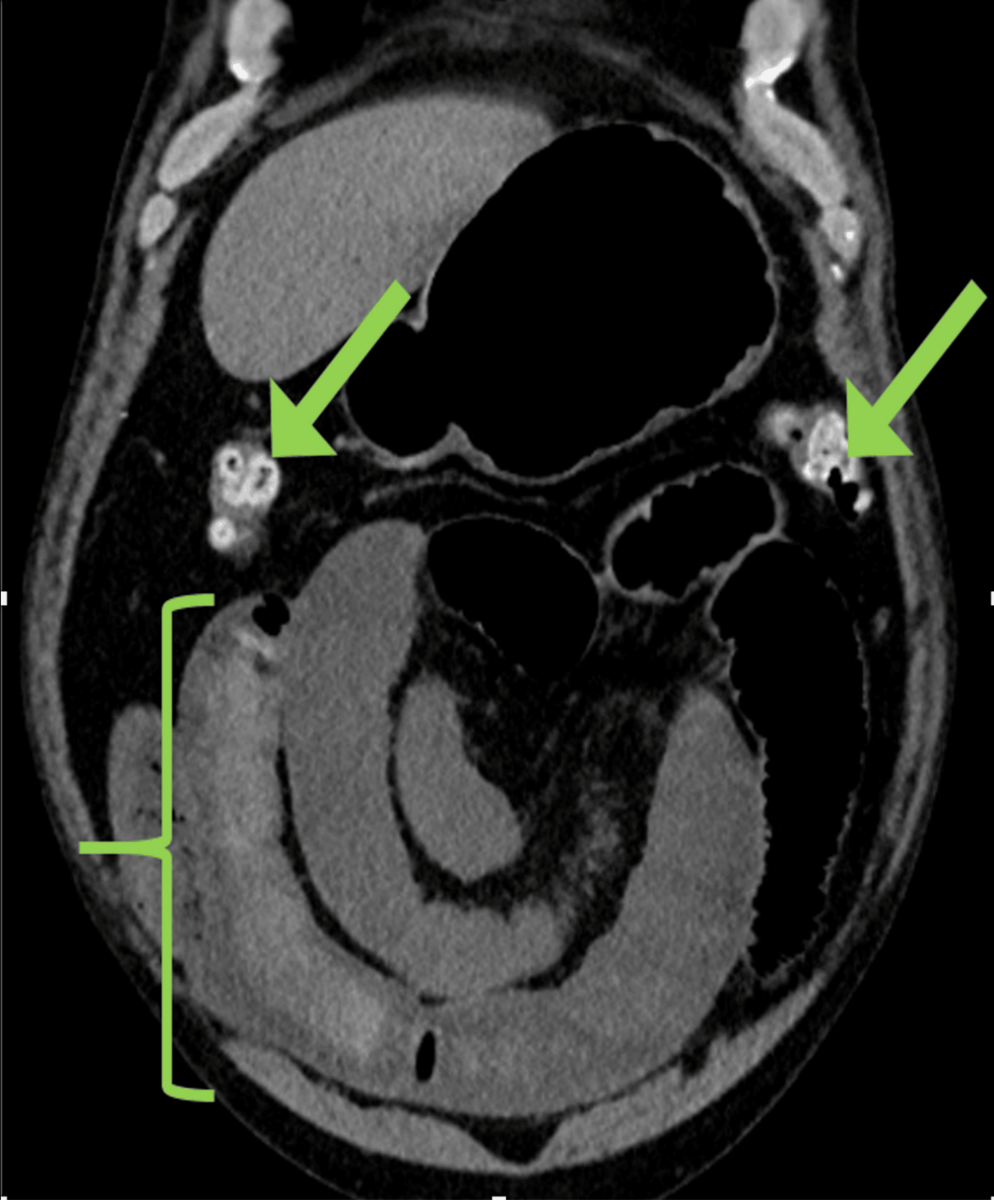
CT coronal imaging, demonstrating colonic scybala associated with opioid-induced chronic constipation (arrows) and residual distal ileal intraluminal barium after modified barium swallow (bracket)

In the ED, the patient was hemodynamically stable. A CT scan of the abdomen revealed a small bowel obstruction with a transition point in the terminal ileum (Figure 4). He underwent non-operative management with intestinal decompression via a nasogastric tube. However, the patient rapidly deteriorated, developing peritonitis, and was taken for emergency exploratory laparotomy.

**Figure 5:**
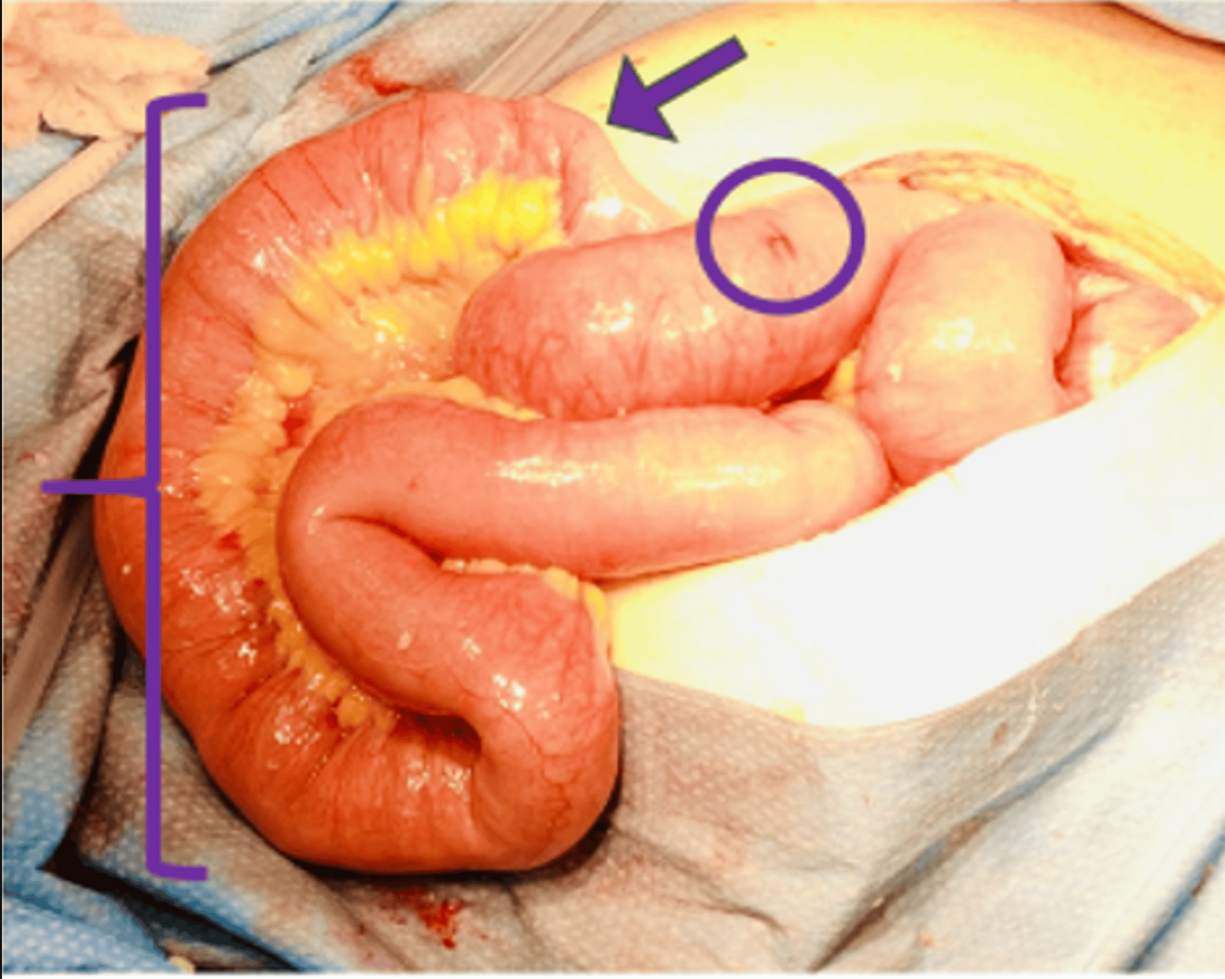
Reversible mesenteric ischemia (circle) proximal to terminal ileal intraluminal chemobezoar (bracket) and small bowel obstruction transition point at the rounded end of intraluminal mass (arrow)

**Figure 6:**
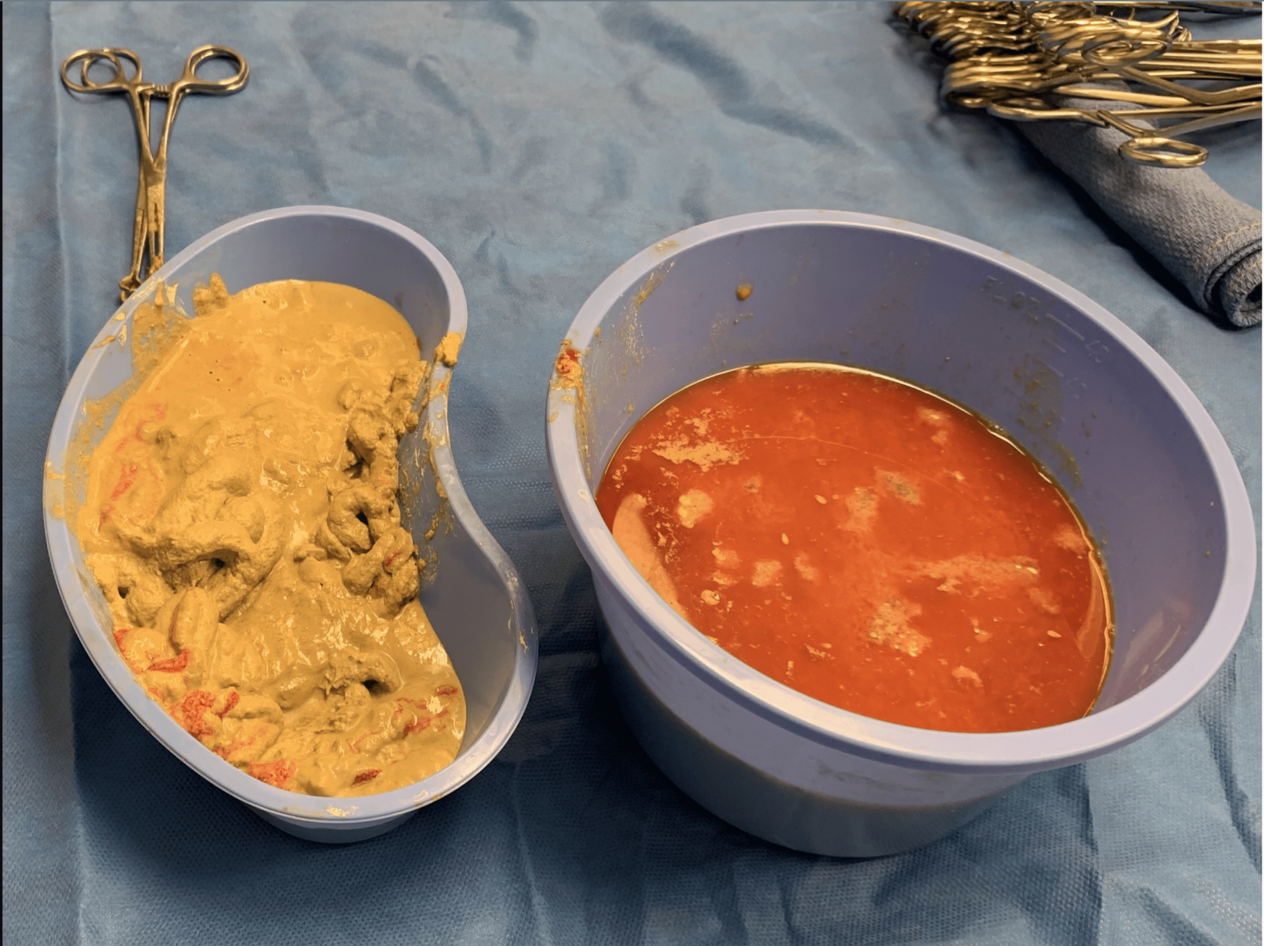
Dense barium chemobezoar to the left evacuated from the lumen with proximal ileal fluid to the right for comparison

Intraoperatively, he was found to have reversible mesenteric ischemia with an obstructive chemobezoar of inspissated bile and stool in the terminal ileum (Figure 5). There were also multiple mobile scybala (hardened fecal masses) throughout the collapsed colon. Bowel contents were emptied both retrograde and antegrade via an enterotomy created proximal to the chemobezoar. A dense barium chemobezoar evacuated from the lumen compared with proximal ileal fluid is shown for comparison (Figure 6).

The patient required ICU-level care post-operatively, including mechanical ventilation, vasopressors, and critical care fluid management. Pathological analysis confirmed impacted material admixed with inspissated barium. Recovery of bowel function was accelerated using methylnaltrexone to relieve opioid-related constipation and gastrografin administration through the nasogastric tube to assist in the evacuation of the scybala. The patient ultimately recovered well and was discharged from the hospital on post-operative day seven.

## Discussion

As barium chemobezoars are rarely reported, their diagnosis requires a heightened awareness of clinical presentation and early identification of the barolith on imaging. Baroliths may present as either small or large bowel obstructions and can appear days to weeks following the use of barium sulfate in imaging. Initial management includes supportive care, such as stool softening agents, laxatives, hydration, and bowel rest with tube decompression. However, as seen in our cases, obstruction due to a barolith can lead to significant morbidity with a risk of bowel necrosis, necessitating operative intervention.

Most cases of barium chemobezoars present with obstructions that form in the rectosigmoid colon [1]. The location of lodgment may depend on gut motility and any additional medical comorbidities that impede normal intestinal transit. In our second case, the patient had a known risk factor of chronic opioid-related constipation, which likely contributed to the increased risk of proximal intestinal obstruction observed at sites of decreased luminal diameter, commonly at the terminal ileum and ileocecal valve.

Published case reports dominate the literature on barium chemobezoars [2-4]. Reports of barium-induced constipation date back to the late 1990s, including a 72-year-old male patient who presented with severe constipation and an abdominal mass 10 days after undergoing a barium enema study for chronic constipation [2]. The diagnosis of a colonic barium chemobezoar was made after exploratory laparotomy, followed by a total colectomy with ileo-rectal anastomosis. One case report of small bowel obstruction (SBO) details a 55-year-old Japanese male patient who presented with abdominal pain, distension, and nausea after an upper gastrointestinal series for gastric cancer screening. The patient required surgical removal of the barium [3]. Another report describes barium-induced constipation in a 64-year-old female patient with diarrhea and overflow incontinence as a result of impacted stool, which required disimpaction under anesthesia [4]. Like previously reported cases, our patients required operative intervention [1]. Both patients developed critical illness, and to our knowledge, these are the first reported cases of barium-induced SBO requiring critical care.

Correlation with recent diagnostic imaging studies using barium ingestion should raise clinical suspicion for the possibility of barium-induced bowel obstruction. As barium sulfate is insoluble, it lines the intestinal lumen and may form a clear point of illuminated obstruction (Figure 1). This obstruction may have a delayed presentation as the barium deposits within fecal content and forms a hardened, well-defined heterogeneous mass within the intestine. Bowel obstruction becomes evident with proximal intestinal dilatation and downstream or distal intestinal collapse (Figure 1).

Slowed transit of the barium may lead to gradual inspissation of the chemical, and rather than a scybala-like image, the contrast may fill the entire lumen, appearing more liquid than solid (Figure 2). Despite the benign appearance of this fluid-filled lumen, the actual density of the barium chemobezoar in the setting of decreased bowel motility prevented the progress of the chemobezoar, resulting in further inspissation before obstruction (Figure 3).

For patients with a history of chronic opioid use, alternative diagnostic studies for dysphagia other than MBS may be considered due to the risk of barium-induced bowel obstruction. Alternatives for dysphagia work-up that do not require barium ingestion include endoscopy, manometry, pH monitoring, and plain radiographs [5]. Endoscopy can be both diagnostic and therapeutic in cases of esophageal stricture and impacted food bolus. It also allows for real-time biopsies, offering more cost-effectiveness [6]. Manometry may be useful but is often poorly tolerated and is less effective for detecting abnormalities than the aforementioned methods. pH monitoring can also be used; however, it is mainly the gold standard for diagnosing reflux-related disorders and may not be as useful for measuring aspiration risk. Lastly, plain radiographs of the neck and chest can detect structural abnormalities contributing to dysphagia.

Although barium studies are part of the work-up for dysphagia and other digestive tract disorders, we recommend that such studies be avoided in patients with risk factors for intestinal dysmotility. In addition to opioid dependence, other risk factors include a history of chronic constipation, neurological disease, and certain medications, such as diuretics, antidepressants, antihistamines, anticonvulsants, and aluminum antacids [7].

## Conclusions

The two cases highlight the diverse clinical contexts in which barolith-related bowel obstruction and life-threatening complications of this disease can arise, emphasizing the importance of high clinical suspicion and prompt intervention. Currently, there are no established guidelines for diagnosing and managing obstructive baroliths, underscoring the need for a multidisciplinary approach involving clinicians, radiologists, and surgeons. Early recognition based on detailed patient history and imaging studies is crucial, as delayed intervention can lead to severe morbidity or mortality. Further research and consensus in this area are warranted to improve outcomes for patients with obstructive baroliths.
